# Low‐dose aspirin can inhibit exosomal release induced by radiotherapy in breast cancer and attenuate its inhibitory effect on NK cell proliferation

**DOI:** 10.1002/cam4.6274

**Published:** 2023-07-01

**Authors:** Li Wang, Zaoxiu Hu, Ceshi Chen, Ting Chen, Zhihong Yao, Wenhui Li, Zuozhang Yang

**Affiliations:** ^1^ Department of Radiotherapy Third Affiliated Hospital of Kunming Medical University (Yunnan Cancer Hospital, Yunnan Cancer Center) Kunming China; ^2^ Department of Pathology Third Affiliated Hospital of Kunming Medical University (Yunnan Cancer Hospital, Yunnan Cancer Center) Kunming China; ^3^ Key Laboratory of Animal Models and Human Disease Mechanisms of Chinese Academy of Sciences and Yunnan Province Kunming Institute of Zoology Kunming China; ^4^ Department of Nuclear Medicine Third Affiliated Hospital of Kunming Medical University (Yunnan Cancer Hospital, Yunnan Cancer Center) Kunming China; ^5^ Bone and Soft Tissue Tumors Research Center Third Affiliated Hospital of Kunming Medical University (Yunnan Cancer Hospital, Yunnan Cancer Center) Kunming China

**Keywords:** aspirin, breast cancer, exosomes, natural killer cells, radiotherapy

## Abstract

**Background:**

Breast cancer (BC) seriously threatens women's health. Aspirin plays a key role in the treatment and prognosis of BC.

**Objective:**

To explore the effect of low‐dose aspirin on BC radiotherapy through the mechanism of exosomes and natural killer (NK) cells.

**Methods:**

BC cells were injected into the left chest wall to establish a BC model in nude mice. Tumor morphology and size were observed. Immunohistochemical staining for Ki‐67 was used to observe the proliferation of tumor cells. TUNEL was used to detect the apoptosis of cancer cells. Protein levels of exosomal biogenesis‐ and secretion‐related genes (Rab 11, Rab27a, Rab27b, CD63, and Alix) were detected by Western blot. Flow cytometry was used to detect apoptosis. Transwell assays were used to detect cell migration. A clonogenic assay was used to detect cell proliferation. Exosomes of BT549 and 4T1‐Luc cells were extracted and observed by electron microscopy. After the coculture of exosomes and NK cells, the activity of NK cells was detected by CCK‐8.

**Results:**

The protein expression of genes related to exosomal genesis and secretion (Rab 11, Rab27a, Rab27b, CD63, and Alix) in BT549 and 4T1‐Luc cells was upregulated under radiotherapy treatment. Low doses of aspirin inhibited exosome release from BT549 and 4T1‐Luc cells and alleviated the inhibitory effect of BC cell exosomes on NK cell proliferation. In addition, knocking down Rab27a reduced the protein levels of exosome‐related and secretion‐related genes in BC cells, further enhancing the promotive effect of aspirin on NK cell proliferation, while overexpressing Rab27a had the opposite effect. Aspirin was combined at a radiotherapeutic dose of 10 Gy to enhance the radiotherapy sensitivity of radiotherapy‐tolerant BC cells (BT549R and 4T1‐LucR). Animal experiments have also verified that aspirin can promote the killing effect of radiotherapy on cancer cells and significantly inhibit tumor growth.

**Conclusion:**

Low doses of aspirin can inhibit the release of BC exosomes induced by radiotherapy and weaken their inhibition of NK cell proliferation, promoting radiotherapy resistance.

## 1 INTRODUCTION

Breast cancer (BC) is one of the most common malignant tumors that harm women's health, and it is an important factor that seriously affects women's lives and quality of life.^1^ At present, the morbidity and mortality rates of BC in China are on the rise, and the incidence of BC is becoming more prevalent in the younger population.^2^ To date, chemotherapy, surgery, radiotherapy, endocrine therapy, and molecular‐targeted therapy have been used to treat BC. Among them, radiotherapy can help reduce the risk of recurrence of breast tissue and adjacent lymph node tumors and reduce the case fatality rate of patients. However, early endogenous radioresistance and acquired radioresistance during radiotherapy are the current challenges and obstacles for BC patients treated with radiotherapy.^3^, ^4^ Therefore, in‐depth study of the molecular mechanism of BC radioresistance can provide new ideas for improving the treatment strategy of BC, improve the clinical outcome, and provide a theoretical basis for the application of radiotherapy in the treatment of diseases.

To escape the recognition and attack of the immune system, cancer cells can grow and metastasize under the control of some mechanisms, which is also called tumor immune escape and is an important strategy for the survival and development of cancer and one of the difficulties in curing cancer.^5^ Tumor immune escape is induced by many factors, including low immunogenicity of tumor cells, recognition of tumor‐specific antibodies as autoantigens, regulation of tumor surface antigens, tumor‐induced immune regions, and tumor‐induced immunosuppression, among which tumor‐induced immunosuppression is the most widely studied mechanism.^6^ Natural killer (NK) cell therapy is a therapeutic strategy aimed at the immune escape mechanism of tumor cells,^7^ and it can produce antitumor effects without causing graft versus host disease.^8^ Therefore, increasing attention has been given to the use of NK cells for tumor immunotherapy. For example, Nguyen et al. found specific NK cell‐mediated tumor‐killing activity as well as increased levels of tumor and NK cell‐derived chemokines and cytokines in their studies of immune function in physiological systems. In studies of BC, NK cells were found to become dysfunctional during cancer, and transforming growth factor beta contributed to the metabolic dysfunction of circulating NK cells in metastatic BC.^9^ Many studies have shown that NK cells have great potential in the treatment of liver cancer, colorectal cancer (CRC), ovarian cancer, BC, non‐small cell lung cancer, and other solid tumors.^10^

At present, the mechanisms of tumor metastasis and evasion of immune surveillance are mainly focused on the influence of the tumor microenvironment on tumor immune tolerance, especially the role and mechanism of exosomes.^11^ Innate and adaptive immune cells, including dendritic cells, macrophages, NK cells, and lymphocytes, can all produce exosomes and participate in the life processes of cell growth, signal transmission, and homeostasis.^12^ In addition, studies have shown that the pleiotropic effects of mesenchymal stem cells, particularly their immunomodulatory potential, can be largely attributed to paracrine factors, of which exosomes are one of these paracrine mediators.^13^, ^14^ Exosomes play a key role in regulating inflammation,^15^ and there is growing evidence that exosomes produced by tumor cells can regulate innate immune signaling and responses.^16^ In the study of immunosuppression and immunotherapy, it has been found that tumor‐derived exosomes (TEX) can inhibit an increase in immune cells, inhibit the activity of NK cells, interfere with the differentiation of monocytes, and create a favorable microenvironment for tumors.^17^ Reza Rahbarghazi et al. also reported that tumor‐derived extracellular vesicles (EVs), metastatic microRNAs (miRNAs), and other nucleic acids participate in creating a favorable tumor environment.^18^ EVs are a heterogeneous population of membrane vesicles released from tumor cells that can induce immunosuppressive states by modifying the premetastatic microenvironment by different pathways.^19^ Moreover, Yang et al.^20^ found that exosomal miR 92a‐3p derived from highly metastatic cancer cells promotes epithelial‐mesenchymal transition (EMT) and metastasis by regulating the PTEN/Akt pathway in hepatocellular carcinoma (HCC). Studies have shown that exosomes induce immunosuppression and immune clearance by analyzing the relationship between exosomes and immunity. As a result, the prognosis of patients is not good.^21^ In addition, extensive evidence indicates that the amount of exosomes secreted by tumor cells varies with cellular stress induced by anticancer therapy, leading to the transfer of drug‐resistance phenotypes between BC tumor cells.^22^, ^23^ In tumor progression and therapy, exosomes from BC cells are found to contribute to cancer cell proliferation, metastasis, angiogenesis, chemoresistance, and radioresistance, leading to metastasis of cancer cell lesions to other organs.^24^ Studies have reported that TEX are a novel cancer driver, and targeting exosomes may represent a novel approach to cancer treatment.^25^ Exosomes show great promise in new treatment strategies for diseases.^26^ Therefore, inhibition of exosomal release from BC and enhancement of NK cell metabolic function may be a promising new therapeutic strategy for BC.

As a nonsteroidal anti‐inflammatory drug, aspirin can inhibit cyclooxygenase (COX)‐1 and/or COX‐2 activity and acts as an antiplatelet agent at low doses (75–100 mg) by covalently modifying COX‐1 expressed in mature platelets. It takes higher doses of aspirin (650–1300 mg) to cause COX‐2 acetylation in inflammatory cells, thereby causing its analgesic and anti‐inflammatory effects.^27^ Today, aspirin has chemopreventive properties against cancer in addition to its antipyretic, analgesic, and anti‐inflammatory effects.^28^ For example, Drew and Chan^29^ reported on colorectal tumors and found that aspirin showed certain advantages in preventing adenomatous polyps (adenomas) and most precursor lesions of CRC. In a study of liver cancer,^30^ aspirin was found to have beneficial effects on HCC as a chemopreventive or adjuvant chemotherapy drug. It has been confirmed that radiotherapy may regulate aspirin in tumor cells by changing the activity of nuclear transcription factor (NF‐κB), while aspirin is an inhibitor of NF‐κB, and aspirin can induce apoptosis of cancer cells through NF‐κB activity in CRC and osteosarcoma.^31^, ^32^ In studies with low doses of aspirin (≤100 mg), long‐term use of aspirin was found to reduce the risk of metastasis and recurrence of esophageal, endometrial, gastric, and BC cancer.^33^, ^34^, ^35^ In obesity‐related BC cell studies, high doses of aspirin were found to show significant cytotoxicity, while 2 mM aspirin did not affect the number of 3T3‐L1 adipocytes.^36^ Therefore, we postulate that low doses of aspirin may increase cancer cell radiosensitivity and reduce cancer metastasis and recurrence probability during treatment in BC.

In summary, further exploration of the effect of low‐dose aspirin on the radiosensitivity of cancer cells to explore the role and mechanism of aspirin in exosomes and NK cells under radiotherapy conditions can better provide a theoretical basis for the combination of drugs and radiotherapy in the treatment of cancer.

## 2 MATERIALS AND METHODS

### 2.1 Construction of BC nude mouse model

Thirty 42‐day‐old female nude mice (18–22 g) were obtained from the Animal Experimental Center of Kunming Medical University for the study. All mice were kept under standard conditions of humidity (50 ± 5%), appropriate temperature (25°C), evenly distributed light and dark conditions, standard diet, free drinking water, and 12 h fasting without water deprivation before operation. All experimental animals were given humane care in accordance with the 3 R principle, and the protocol was implemented in accordance with the provisions of the China Animal Science Standardization Technical Committee.

The BC nude mouse model was established according to the literature.^37^ The mice were randomly divided into five groups (*n* = 6 mice/group): BC group, BC + 10 Gy group, BC + 10 Gy + aspirin group, BC‐R (BC tolerant cell line) + 10 Gy group, and BC‐R + 10 Gy + aspirin group. The BC group was treated as follows. BT‐549 (human mammary ductal carcinoma cell) (Punosai) was digested and washed twice with PBS to remove the residual medium. The cell density was kept to 5 × 10^6^/mL, and 0.1 mL was subcutaneously injected into the left chest wall of nude mice, and the state of nude mice and the growth of tumors were observed weekly. The BC + 10 Gy group was treated as follows. The nude mice were inoculated with BT‐549 cells for 2–3 weeks and were irradiated twice a week with 0.1 Gy/min for 10 min. The BC + 10 Gy + aspirin group was treated as follows. The nude mice were inoculated with BT‐549 cells for 2–3 weeks, and 25 mg/kg/d aspirin was given to the nude mice by gavage. At the same time, the nude mice were irradiated twice a week with 0.1 Gy/min of total body irradiation for 10 min. The BC‐R + 10 Gy group was treated as follows. When nude mice were inoculated with tolerant BT‐549 (BT‐549 R) cells for 2–3 weeks, the whole body of nude mice was irradiated with 0.1 Gy/min for 10 min per week, and the radiotherapy was conducted twice a week. The BC‐R + 10 Gy + aspirin group was treated as follows. Twenty‐five mg/kg/d aspirin was intragastrically administered to the nude mice 2–3 weeks after the inoculation of BT‐549 R cells, and the whole body of the nude mice was irradiated twice a week with 0.1 Gy/min for 10 min. Tumor size was measured with calipers, and all experimental nude mice were euthanized with an overdose of sodium pentobarbital on Day 21 after tumor inoculation.

### 2.2 Immunohistochemistry

After euthanasia, the tumor tissues of the nude mice were quickly collected, fixed with 4% paraformaldehyde, embedded in paraffin, dehydrated with a concentration gradient, and cut into 5 μm thick sections using a Leica paraffin microtome. After routine dewaxing, antigen retrieval and blocking were performed, followed by overnight incubation with Ki‐67 antibody (Abcam, 1:1000) in a humidified environment at 4°C. In the dark, the mixture of the sample and the secondary antibody was cultured at 25°C for h. The developed film was rinsed with clean water for a period of time, sealed with neutral gum (Shanghai Biyuntian Co., Ltd.), and photographed with an Olympus BX51 microscope.

### 2.3 Terminaldeoxynucleotidyl transferase‐mediated dUTP nick end labeling (TUNEL) staining

A TUNEL apoptosis detection kit (Elabscience) was used to detect cancer cell apoptosis in tissues. Tissue slices were routinely dewaxed and then washed twice with xylene and once with gradient ethanol (100%, 95%, 90%, 80%, 70%). Proteinase K working solution was used to treat the tissues, and PBS was used to rinse the tissues twice. The TUNEL reaction mixture was added, dehydrated with ethanol, cleared with xylene, and sealed with neutral gum. A drop of PBS or glycerol was added under the visual field, the apoptotic cells were observed with a light microscope, and pictures were taken.

### 2.4 Cell culture and construction of radiotherapy tolerance

The human BC BT549 cell line (Punosai) and mouse BC cell line 4T1‐Luc (Punosai) were cultured with RPMI‐1640 containing 10% FBS and 1% P/S in an incubator at 37°C and 5% CO_2_. Exponential BT549 and 4T1‐Luc cells were transferred to a 6 cm^2^ flask, which was placed on a linear accelerator PRIMUS‐H (Siemens) with a fixed source skin distance, and X‐ray irradiation was performed at 0.6 Gy/min for 10 min. The culture medium was replaced immediately after irradiation, and the culture medium was replaced appropriately according to the growth of cells during the culture process. When the cells grew close to the bottom of the bottle, pancreatic enzyme digestion was inoculated and passed, and when the cell proliferation entered the exponential growth stage, X‐ray irradiation was performed again at room temperature at 6 Gy. After 10 repeated irradiation cycles, the cells were subcultured until the cell morphology and proliferation status were stable, and then the BT549R and 4T1‐LucR cell lines were screened.

### 2.5 Radiotherapy process

The cells were digested with trypsin 12 h before radiotherapy, centrifuged at 800 r/min for 5 min, and then resuspended. Appropriate and equal amounts of cells were evenly inoculated into the culture dish so that the confluence rate of the cells was approximately 70%. RPMI‐1640 was used for culture and incubated in a constant temperature incubator. Then, the adherent and stable growth cells were removed from the incubator, and the medium was replaced after PBS cleaning. The edge was sealed with a sealing film, and the medium was placed under the irradiation head of the linear accelerator for dose irradiation of 0, 2, 4, 6, 8, and 10 Gy (0.6 Gy/min). After irradiation, the sealing film was removed, and the culture was continued for 48 h in a constant temperature incubator.

### 2.6 Cell transfection and drug treatment

The cells were subcultured and inoculated on a 6‐well plate. si‐Rab27a and oe‐Rab27a were transfected into the cells using a Lipofectamine 2000 kit (Solebo) when the cell growth density was approximately 50% and cultured in a 5% CO_2_ cell incubator at 37°C.

DMSO was used to dissolve aspirin at a concentration of 2 mM. The control group was treated with the same volume of DMSO as aspirin, while the experimental group was treated with 2 mmol/L aspirin.^28^ Each group was set up with 5 duplicate wells for continuous treatment for 48 h. The cells were then cultured in a 37°C, 5% CO_2_ cell culture incubator.

### 2.7 Observation of exosomes by transmission electron microscopy

The culture supernatants of the third to sixth passages of BT549 and 4T1‐Luc cells were collected, and 1.2 mL was added to a 100 kD ultrafiltration centrifuge tube, the tube was centrifuged at 4000 × ***g*** for 0.5 h, PBS at pH 7.4 was added, and the tube was gently pipetted to obtain approximately 120 mL of exosome‐containing ultrafiltration concentrate. The above suspension was subjected to gradient centrifugation according to the following steps: centrifugation at 300 × ***g*** for 10 min, centrifugation at 2000 × ***g*** for 10 min, centrifugation at 10,000 × ***g*** for 0.5 h, and centrifugation at 100,000 × ***g*** for 70 min 2 times. The light yellow precipitate attached to the bottom wall of the test tube was the exosome. The exosomes were gently and thoroughly blown and mixed with 1 mL PBS to fully reinsert them. Then, 10 μL drops on the 200‐mesh carbon‐film copper mesh were added, and the samples were left on the ultraclean table to ventilate for 15 min. After, a sterile clean filter paper was used to absorb the excess liquid, and then 1% uranyl acetate was added for negative staining for 5 min, a sterile clean filter paper was used to absorb the excess negative staining solution, and the samples were allowed to stand for 0.5 h and dry at room temperature. The morphology and size of exosomes were observed by transmission electron microscopy, and the unused exosomes were stored at −80°C for later use.

### 2.8 Identification and culture of NK cells

NK cells (Punosai) were resuscitated and inoculated in a 6‐well cell culture plate. After trypsin digestion, an appropriate amount of NK cells was taken, the supernatant was centrifuged and discarded, PBS was added to resuspend the cells, and then FITC‐labeled antibodies were added and incubated at room temperature away from light. NK cell surface markers were detected on the machine. The NK cells were subcultured on a 6‐well plate. When the cell growth density was approximately 50%, the exosome suspension extracted from the cells in each group was aspirated into the cell culture plate, and the cells were cultured at 37°C and 5% CO_2_.

### 2.9 CCK8 detection of cell proliferation

In this study, we aimed to determine neuronal cell viability, and a CCK‐8 kit (Solabao) was used. In 96‐well plates, cells were cultured with intervention in an incubator (37°C, 5% CO_2_) for preculture. To find the drug concentration, the EC solution was added in the order of 0, 100, 200, 300, and 400 μL in a concentration gradient. EC solution was added sequentially in a concentration gradient. In an incubator (37°C), 100 μL of the medium was added and cultured for 1 day. After transfection or dosing, 10 μL of CCK‐8 reagent were added and cultured for 2 h. Finally, an enzyme marker (ELX800, BioTeK) was used to measure the absorbance at 450 nm.

### 2.10 Western blot protein levels were measured

After quantitative analysis, the total protein of the sample was extracted and denatured in this study. SDS–PAGE gel was used for electrophoresis, electrophoresis apparatus (Bio‐RAD) was adjusted to 120 V for electrophoresis, PVDF membrane (Millipore) was used for membrane transfer, and skim milk (Sigma) was used for blocking. Primary antibodies (Abcam) were then added and incubated overnight. The antibodies used were as follows: anti‐Rab11 (Abcam, 1:1000), anti‐Rab27a (Abcam, 1:200), anti‐Rab27b (Abcam, l:500), anti‐TSAP6 antibody (Abcam, 1:1000), anti‐CD63 antibody (Abcam, 1:1000), anti‐CD9 antibody (Abcam, 1:1000), anti‐Alix antibody (Abcam, 1:1000), and anti‐β‐Actin antibody (Abcam, 1:10,000). The membrane was then incubated with the primary antibody diluted in advance. Subsequently, the membranes were incubated with IgG (Abcam, 1:300) for 1 h. Finally, the protein bands were visualized using a Pierce ECL Western blotting kit. Protein expression was quantified using ImageJ to analyze band gray values. β‐Actin was used as a control.

### 2.11 The apoptosis rate was detected by flow cytometry

Cultured cells were collected into cell suspensions using trypsin digestion and washed twice with cold PBS. According to the instructions for the Apoptosis Assay Kit (Solebo), 5 μL Annexin  V‐FITC was added to the cell suspension. In the dark, the solution was mixed well and incubated at 25°C for 15 min, and 5 μL of PI staining solution was added, mixed, and incubated for 5 min. Finally, the level of apoptosis was detected by flow cytometry.

### 2.12 Transwell chamber assays cell migration

A 24‐well Transwell chamber (Beijing Unikon Biotechnology Co., Ltd.) was used to determine cell migration. Transfected cells were collected 48 h after transfection, suspended in FBS‐free medium, and cultured in the upper chamber at an initial density of 5 × 10^4^/well. In the lower chamber, 500 μL of medium containing FBS was used as a chemical inducer. Then, the cells that did not migrate were carefully removed with a cotton swab after incubation at 37°C for 2 days. Subsequently, the migrated cells were fixed with 95% methanol at 25°C for 15 min, and 0.1% crystal violet‐formalin solution was used for staining for 15 min, and images were captured with an IX71 inverted microscope (Shanghai Zeshi Photoelectric Technology Co., Ltd.).

### 2.13 Cell proliferation was detected by cell colony formation assay

In the logarithmic growth phase, cells from each group were treated with 0.25% trypsin protease. After digestion, the cell suspension was diluted by gradient, and the cells in each group were inoculated into the dishes containing 10 mL of 37°C preheated culture medium at the gradient density of 50, 100, and 200 cells per dish, respectively. The cell culture was incubated for 2–3 weeks. Cultures were terminated when macroscopic clones appeared in the dish. Cells were fixed with 5 mL of 4% paraformaldehyde for 15 min. Then, the fixative was removed, an appropriate amount of GIMSA (Solebo) was added, the staining solution was applied for 10–30 min, and then the staining solution was slowly washed away with running water. Clones with more than 10 cells were counted using a microscope.

### 2.14 Statistical analysis

In this study, all experiments in this paper were repeated three times, and for all statistical analyses, GraphPad Prism was used and Student's *t*‐test was used to analyze the data. The statistical significance value was 0.05. The mean value and standard deviation are shown in this study.

## 3 RESULTS

### 3.1 Radiotherapy can inhibit the proliferation of BC cells and increase the release of exosomes from BC cells

**FIGURE 1:**
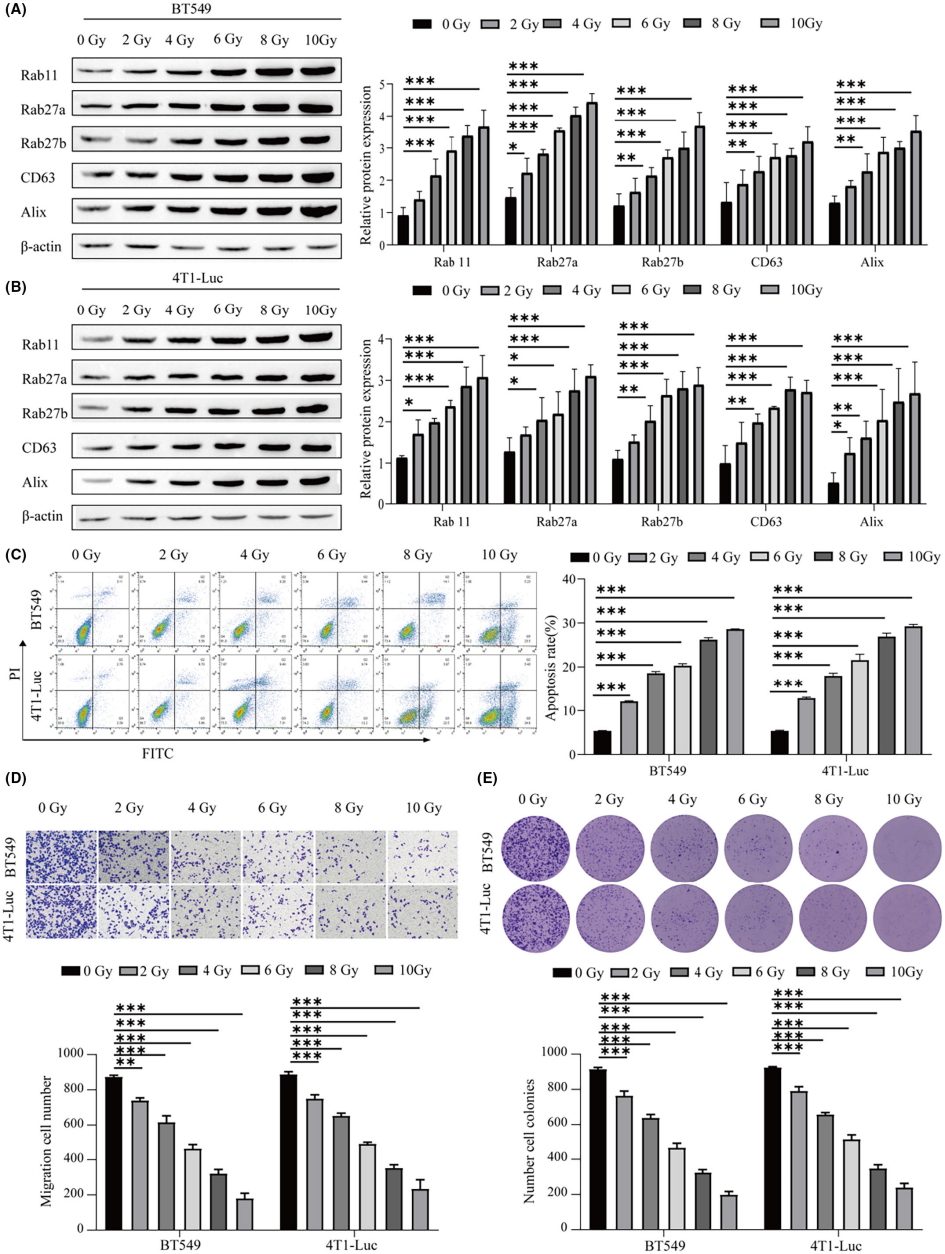
Radiotherapy can inhibit the proliferation of breast cancer (BC) cells and increase the release of exosomes from BC cells. (A) Detection of the protein levels of genes (Rab 11, Rab27a, Rab27b, CD63, and Alix) related to exosome biogenesis and secretion in BT549 cells by Western blot. (B) Detection of the protein levels of genes related to exosomal biogenesis and secretion (Rab 11, Rab27a, Rab27b, CD63, and Alix) in 4T1‐Luc cells by Western blot. (C) Detection of the apoptosis rate by flow cytometry. (D) Transwell was used to detect cell migration. (E) Cell proliferation was measured by cell clonogenic assay. ***p < 0.001, **p < 0.01, *p < 0.05.

First, we treated the BC cell lines BT549 and 4T1‐Luc with different radiotherapy doses. Then, Western blotting was used to detect the protein expression levels of genes related to exosome biogenesis and secretion (Rab 11, Rab27a, Rab27b, CD63, and Alix). The results showed that radiotherapy‐induced the release of exosomes, and the protein expression levels of genes related to exosomogenesis and secretion in BT549 and 4T1‐Luc cells were significantly increased at a dose of 10 Gy compared with the nonirradiated control cells (Figure 1A,B). Using flow cytometry to detect apoptosis, it was found that radiotherapy promoted apoptosis, and the number of apoptotic BT549 and 4T1‐Luc cells was significantly increased at a dose of 10 Gy (Figure 1C). Transwell assays were used to detect the migration of cells, and the migration ability of BT549 and 4T1‐Luc cells decreased with increasing radiotherapy dose (Figure 1D). Moreover, with the increase in radiotherapy dose in the cell clonogenic assay, the proliferation of BT549 and 4T1‐Luc cells decreased (Figure 1E). Therefore, radiotherapy can inhibit cell migration and proliferation, promote cancer cell apoptosis, and increase the release of exosomes from BC cells.

### 3.2 Radiotherapy‐induced release of BC exosomes and inhibition of NK cell proliferation

**FIGURE 2:**
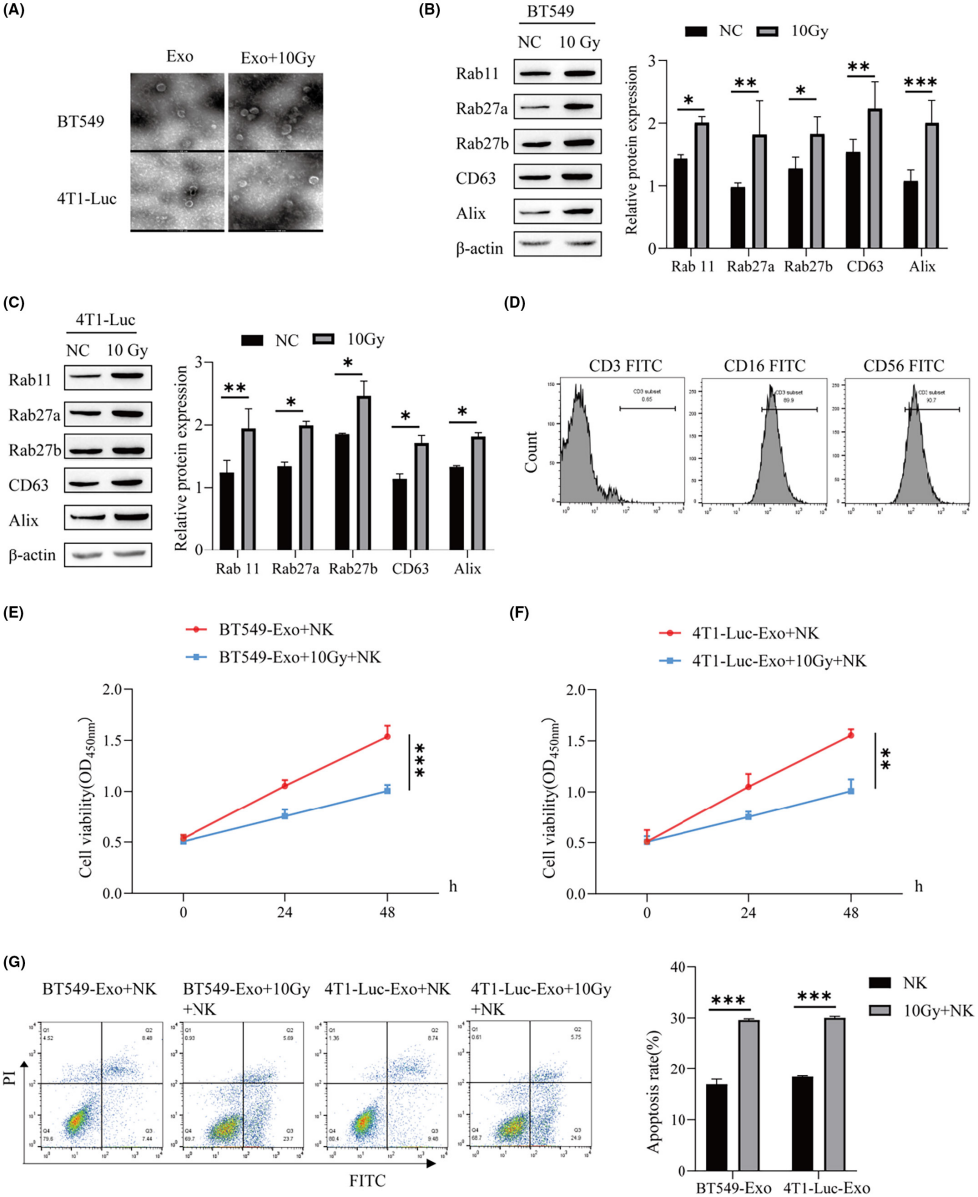
Radiotherapy‐induced release of breast cancer (BC) exosomes and inhibition of natural killer (NK) cell proliferation. (A) The exosomes were observed by electron microscopy. (B) Western blot was used to detect the protein levels of exosomal biogenesis‐ and secretion‐related genes (Rab 11, Rab27a, Rab27b, CD63, and Alix) in BT549 cells. (C) Western blot was used to detect the protein levels of exosomal biogenesis‐ and secretion‐related genes (Rab 11, Rab27a, Rab27b, CD63, and Alix) in 4T1‐Luc cells. (D) Flow cytometry was used to detect NK cell surface markers. (E) CCK‐8 assay was used to detect NK cell activity. (F) CCK‐8 assay was used to determine NK cell viability. (G) Flow cytometry was used to detect the apoptosis rate of NK cells. ***p < 0.001, **p < 0.01, *p < 0.05.

To explore the effect of radiotherapy on the secretion of BC exosomes, we observed the exosomes by electron microscopy, and the secretion of BC exosomes increased after radiotherapy (Figure 2A). Western blotting was used to detect the expression of proteins of exosomal markers of BT549 and 4T1‐Luc cells, and it was found that the protein expression levels of Rab11, Rab27a, Rab27b, CD63, and Alix in the 10 Gy group were significantly higher than those in the NC group (Figure 2B,C). Through flow cytometry, the levels of CD3, CD16, and CD56 were detected, and CD3 was negative, while CD16 and CD56 were positive (Figure 2D). To study the effect of BC exosomes on NK cells. The exosomes of BT549 and 4T1‐Luc cells under normal culture and radiotherapy culture were cocultured with NK cells, and CCK‐8 was used to detect NK cell viability. The cell viability of exosomes irradiated by 10 Gy radiotherapy after coculture with NK cells was lower than that of NK cells cocultured with exosomes under normal culture conditions (Figure 2E,F). Flow cytometry was used to detect NK cell apoptosis. The amount of NK cell apoptosis after coculture of exosomes irradiated by 10 Gy radiotherapy and NK cells was increased in the group without radiotherapy coculture (Figure 2G). It is suggested that BC radiotherapy can increase the release of exosomes and promote the apoptosis of NK cells.

### 3.3 Low doses of aspirin inhibit the release of BC exosomes and promote the proliferation of NK cells

**FIGURE 3:**
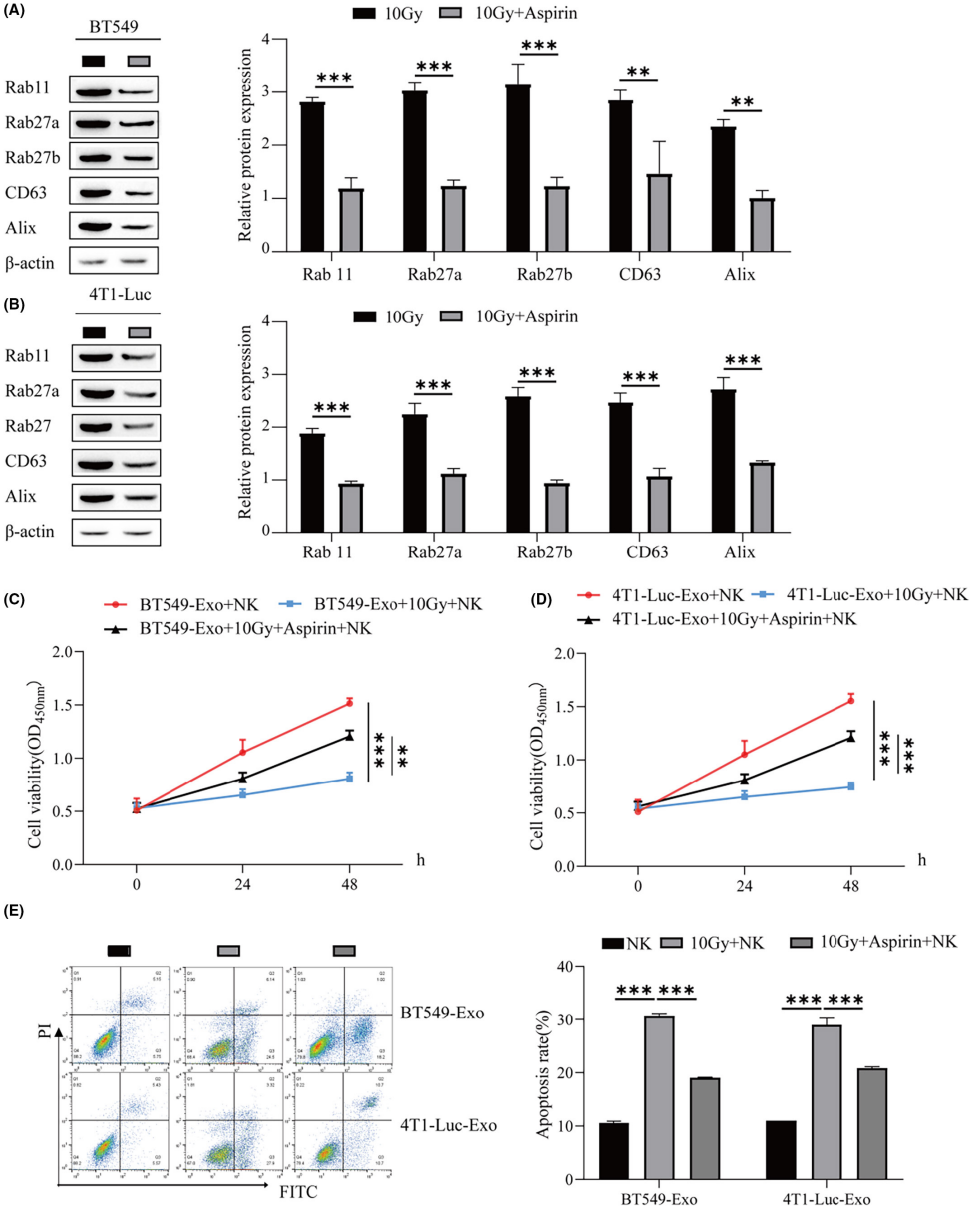
Low doses of aspirin inhibit the release of breast cancer (BC) exosomes and promote the proliferation of natural killer (NK) cells. (A) Western blot was used to detect the protein levels of genes (Rab 11, Rab27a, Rab27b, CD63, and Alix) related to exosome biogenesis and secretion in BT549 cells. (B) Protein levels of genes related to exosomal biogenesis and secretion (Rab 11, Rab27a, Rab27b, CD63, and Alix) in 4T1‐Luc cells by Western blot. (C) CCK‐8 was used to detect NK cell viability. (D) CCK‐8 was used to detect NK cell viability. (E) Flow cytometry was used to detect the apoptosis rate of NK cells. ***p < 0.001, **p < 0.01.

In this study, we used Western blotting to detect the protein levels of genes related to exosome biogenesis and secretion in BT549 and 4T1‐Luc cells. Rab 11, Rab27a, Rab27b, CD63, and Alix protein levels were lower in the 10 Gy + aspirin group than in the 10 Gy group (Figure 3A,B). Aspirin inhibits exosomal release in BT549 and 4T1‐Luc cells. At 2 mmol/L aspirin, the exosomes of BT549 and 4T1‐Luc cells were extracted and cocultured with NK cells after treatment with 10 Gy. The activity of NK cells was detected by CCK‐8. The results showed that the activity of NK cells cocultured with exosomes irradiated by 10 Gy radiotherapy was significantly lower than that of normal cocultured NK cells. The cell viability of exosomes cocultured with NK cells at 10 Gy + aspirin was significantly higher than that of exosomes cocultured with NK cells at 10 Gy (Figure 3C,D). In this study, we used flow cytometry to detect NK cell apoptosis. The apoptosis rate of NK cells cocultured with exosomes at 10 Gy + aspirin was lower than that of NK cells cocultured with exosomes at a 10 Gy irradiation dose (Figure 3E). In conclusion, a low dose of aspirin can inhibit the exosomal release of BC, promote the proliferation of NK cells, and weaken the inhibitory influence of radiotherapy on NK cell apoptosis.

### 3.4 Rab27a, the key regulator of exosomal secretion, can affect the regulation of NK cells by aspirin

**FIGURE 4:**
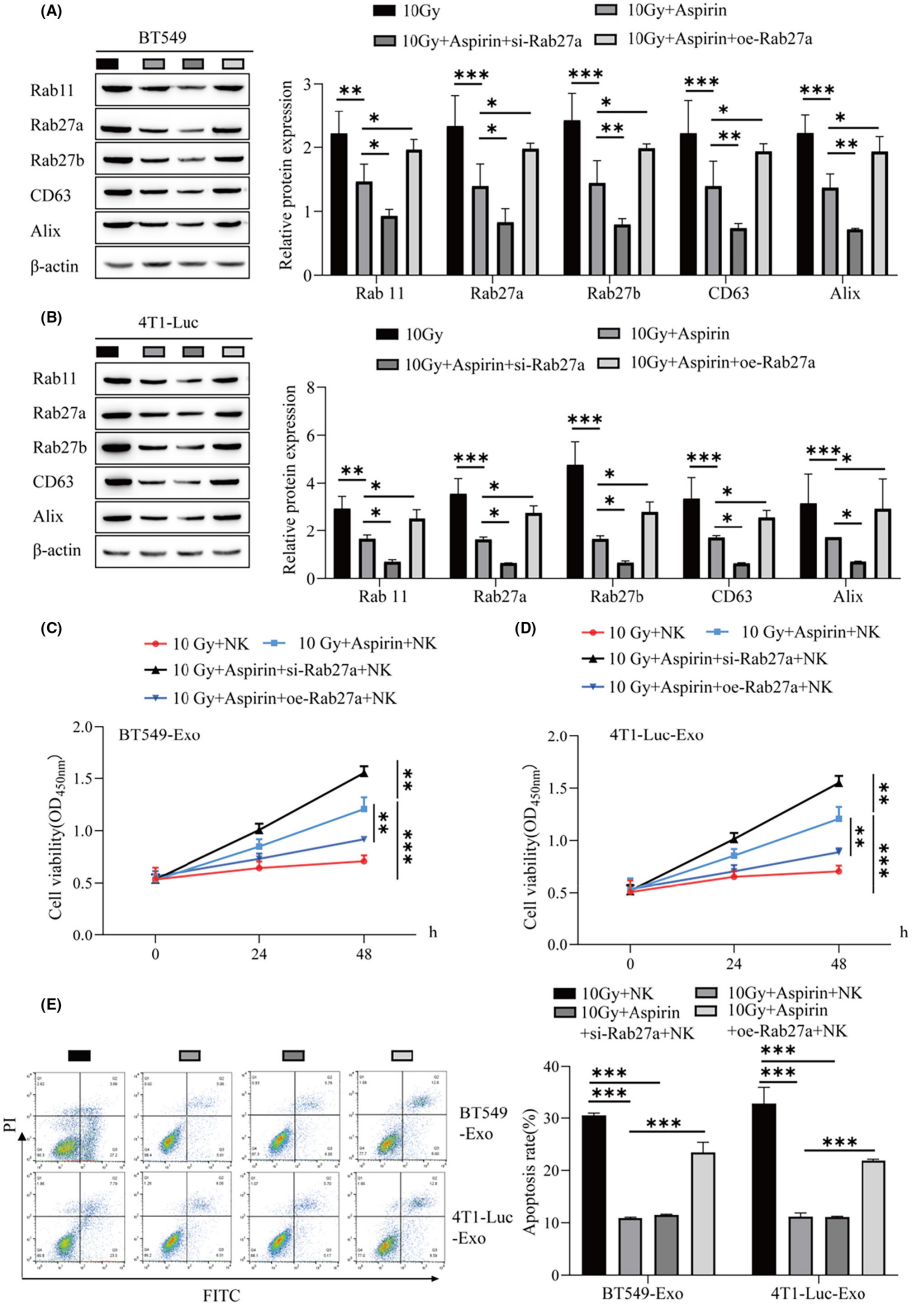
Rab27a, the key regulator of exosomal secretion, can affect the regulation of natural killer (NK) cells by aspirin. (A) Western blot was used to detect the protein levels of genes (Rab 11, Rab27a, Rab27b, CD63, and Alix) related to exosome biogenesis and secretion in BT549 cells. (B) Protein levels of genes related to exosomal biogenesis and secretion (Rab 11, Rab27a, Rab27b, CD63, and Alix) in 4T1‐Luc cells by Western blot. (C) CCK‐8 was used to detect NK cell viability. (D) CCK‐8 was used to detect NK cell viability. (E) Flow cytometry was used to detect the apoptosis rate of NK cells. ***p < 0.001, **p < 0.01, *p < 0.05.

In this study, we used Western blotting to detect the protein levels of exosomal biogenesis‐ and secretion‐related genes in BT549 and 4T1‐Luc cells, and it was found that the addition of 2 mmol/L aspirin inhibited the promotion of radiotherapy on the above proteins, and the knockdown of Rab27a further promoted the effect of aspirin, overexpression of Rab27a reversed the effect of aspirin (Figure 4A,B). BT549 and 4T1‐Luc cells were treated with 10 Gy, aspirin, si‐Rab27a, or oe‐Rab27a, and then the exosomes of BT549 and 4T1‐Luc cells were extracted and cocultured with NK cells, and NK cell activity was detected by CCK‐8. The viability of NK cells cocultured under the condition of 10 Gy + aspirin + si‐Rab27a was higher than that of exosomes cocultured with NK cells under the condition of 10 Gy + aspirin (Figure 4C,D). The NK cell viability of 10 Gy + aspirin + oe‐Rab27a coculture was lower than that of 10 Gy + aspirin exosomes after coculture with NK cells (Figure 4C,D). In this study, we used flow cytometry to detect NK cell apoptosis. The apoptosis rate of NK cells cocultured with 10 Gy + aspirin + oe‐Rab27a was higher than that of NK cells cocultured with exosomes under 10 Gy + aspirin (Figure 4E). In conclusion, a low dose of aspirin can inhibit the exosomal release of BC, promote the proliferation of NK cells, and weaken the inhibitory influence of radiotherapy on NK cell apoptosis. Furthermore, Rab27a knockdown can reduce the expression of genes related to exosomal biogenesis and secretion in cancer cells and promote the proliferation activity of aspirin on NK cells, while Rab27a overexpression can weaken the activity and promote the apoptosis of NK cells.

### 3.5 In vivo experimental verification that aspirin inhibits BC exosomal release and BC progression

**FIGURE 5:**
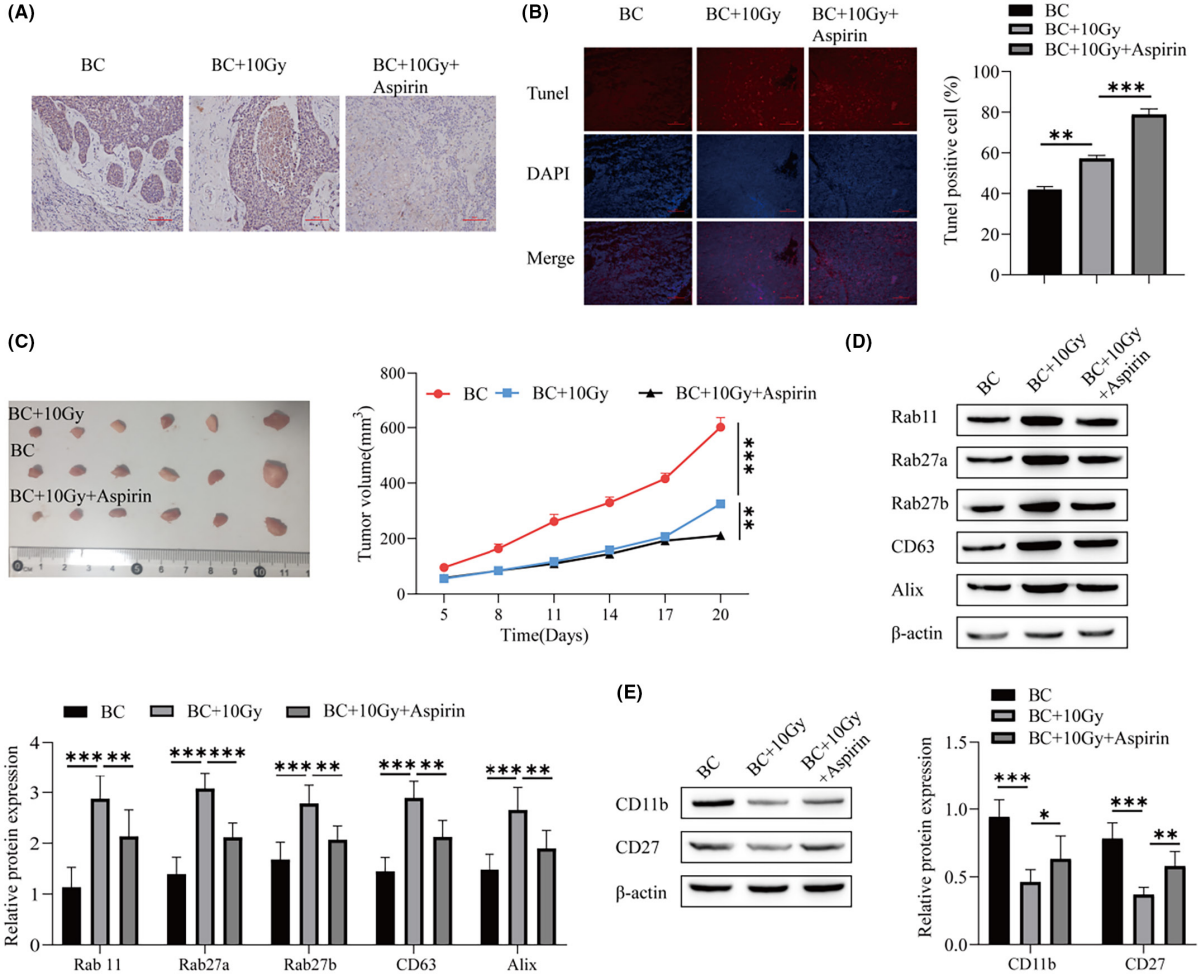
In vivo experiments verify that aspirin inhibits the release of breast cancer (BC) exosomes and the progression of BC. (A) Immunohistochemistry was used to detect the expression of Ki‐67 in tissues. (B) TUNEL was used to detect apoptosis. (C) Nude mouse tumorigenesis experiment. (D) Western blot showing the protein levels of exosomal biogenesis‐ and secretion‐related genes (Rab 11, Rab27a, Rab27b, CD63, and Alix transcripts). (E) Western blot was used to detect the expression of CD11b and CD27. ***p < 0.001, **p < 0.01, *p < 0.05.

In vitro experiments have verified that low doses of aspirin can inhibit the exosomal release of BC and promote its ability to proliferate NK cells. Next, we will explore the effects of low doses of aspirin on cancer cells in animals. Cell proliferation was observed by immunohistochemical detection of Ki‐67. The cell proliferation in the BC + 10 Gy group was lower than that in the BC group, and the combination of aspirin increased the killing effect of radiotherapy on cells (Figure 5A). TUNEL staining indicated that the number of apoptotic cells in the BC + 10 Gy group was higher than that in the BC group, and the number of apoptotic cells in the BC + 10 Gy + aspirin group was significantly higher than that in the BC + 10 Gy group (Figure 5B). The tumorigenesis experiment in nude mice showed that the tumor volume was smaller than that in the BC group at the radiotherapy dose of 10 Gy, while the tumor volume was smaller than that in the BC + 10 Gy group with the addition of aspirin (Figure 5C). Radiotherapy promoted the apoptosis of cancer cells, and its combination with aspirin enhanced the killing effect of radiotherapy on cancer cells. Western blotting was used to detect the protein levels of genes related to the biogenesis and secretion of exosomes, and the protein expression levels of Rab11, Rab27a, Rab27b, CD63, and Alix in the BC + 10 Gy group were higher than those in the BC group. Coadministration of aspirin reduced the promotion of protein levels of genes involved in exosomal biogenesis and secretion at a dose of 10 Gy radiotherapy (Figure 5D). Aspirin was found to impair the inhibitory effect of the 10 Gy radiotherapy dose on CD11b and CD27 (Figure 5E). Aspirin can promote the killing effect of radiotherapy on cancer cells.

### 3.6 Effect of exosomes from radiotolerant BC cells on NK cell proliferation

**FIGURE 6:**
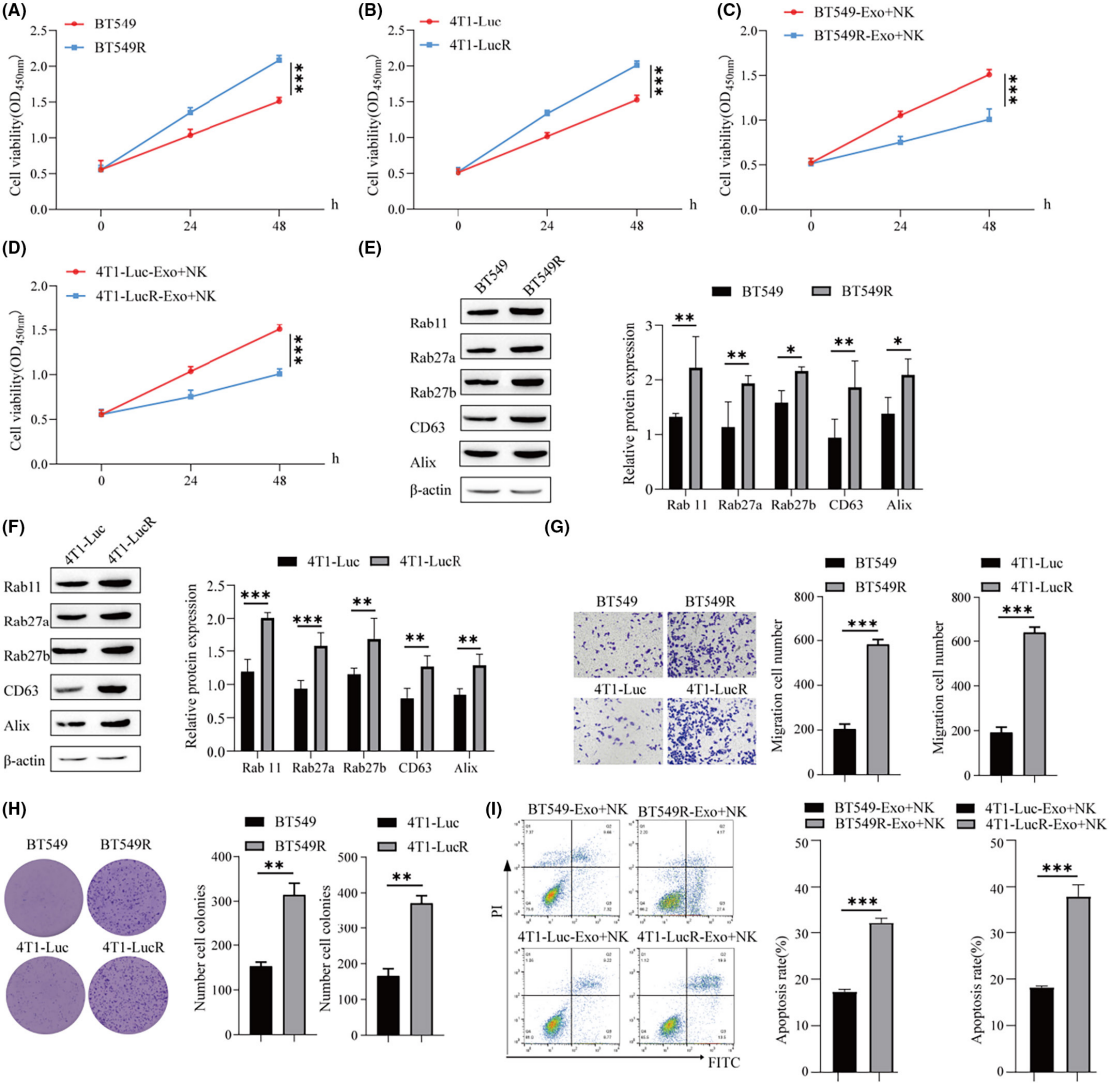
Effect of exosomes from radioresistant breast cancer (BC) cells on natural killer (NK) cell proliferation. (A–D) CCK‐8 assays were used to detect cell viability. (E) Western blotting was used to detect the protein levels of genes involved in exosomal biogenesis and secretion (Rab 11, Rab27a, Rab27b, CD63, and Alix). (F) Western blotting was used to detect the protein levels of genes related to exosomal biogenesis and secretion (Rab 11, Rab27a, Rab27b, CD63, and Alix) in 4T1‐Luc cells. (G) Cell migration was detected by Transwell assay. (H) Cell proliferation was detected by clonogenic assay. (I) Flow cytometry was used to detect the apoptosis rate of NK cells. ***p < 0.001, **p < 0.01, *p < 0.05.

The BC cell lines BT549R and 4T1‐LucR, which are resistant to radiotherapy, were cultured, and CCK‐8 assays showed that the cell viability of BT549R and 4T1‐LucR cells was significantly higher than that of BT549 and 4 T1‐Luc cells (Figure 6A,B). Exosomes were extracted from BT549R and 4T1‐LucR and cocultured with NK cells. The NK cell viability of the coculture was significantly lower than that of the coculture with the exosomes extracted from the intolerant cells (Figure 6C,D). Western blot results showed that Rab11, Rab27a, Rab27b, CD63, and Alix protein expression was higher in BT549R and 4T1‐LucR cells than in BT549 and 4T1‐Luc cells (Figure 6E,F). Transwell analysis of cell migration revealed that BT549R and 4T1‐LucR cells had higher migration capacity than intolerant cells (Figure 6G). The cell colony formation assay showed that the proliferation ability of BT549R and 4T1‐LucR cells was higher than that of intolerant cells (Figure 6H). Exosomes were extracted and cocultured with NK cells, and NK cell apoptosis was detected by flow cytometry. The NK apoptosis rate in the BT549R‐Exo + NK group and the 4T1‐LucR‐Exo + NK group was higher than that in the BT549‐Exo + NK group and the 4T1‐Luc‐Exo + NK group (Figure 6I). In conclusion, radioresistant BC cells were more migration and had a stronger ability to induce NK cell apoptosis.

### 3.7 Aspirin can enhance the sensitivity of radiation‐resistant BC cells to radiotherapy

**FIGURE 7:**
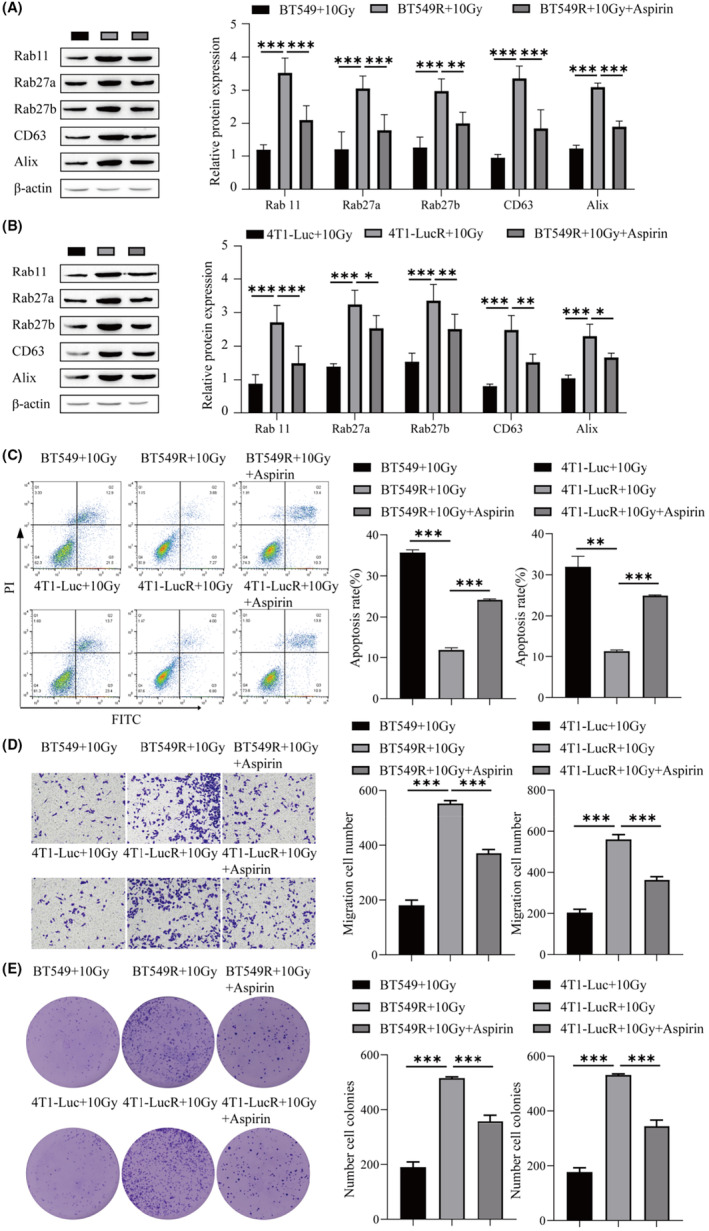
Aspirin can enhance the sensitivity of radiation‐resistant breast cancer (BC) cells to radiotherapy. (A) Western blot was used to detect the protein levels of genes (Rab 11, Rab27a, Rab27b, CD63, and Alix) related to exosome biogenesis and secretion in BT549 cells. (B) Western blot was used to detect the protein levels of genes related to exosomal biogenesis and secretion (Rab 11, Rab27a, Rab27b, CD63, and Alix) in 4T1‐Luc cells. (C) Flow cytometry was used to detect the apoptosis rate. (D) Transwells were used to detect cell migration. (E) Cell clonogenic assay was used to detect cell proliferation. ***p < 0.001, **p < 0.01, *p < 0.05.

The sensitivity of the tolerant strains was lower than that of the intolerant strains. Next, we will verify the effect of aspirin and a 10 Gy radiotherapy dose on the sensitivity of BC‐tolerant strains. Western blot results showed that the protein expression levels of Rab11, Rab27a, Rab27b, CD63, and Alix in BT549R and 4T1‐LucR cells were significantly higher than those in BT549 and 4T1‐Luc cells at 10 Gy. Concomitant use of aspirin attenuated the enhancement of Rab11, Rab27a, Rab27b, CD63, and Alix protein expression by radiotherapy (Figure 7A,B). Flow cytometry showed that the apoptosis rate of BT549R and 4T1‐LucR cells treated with 10 Gy radiotherapy was lower than that of intolerant cells, while the addition of aspirin promoted the apoptosis of BT549R and 4T1‐LucR cells (Figure 7C). The migration ability of resistant cells was higher than that of intolerant cells at a dose of 10 Gy, while the combination of aspirin inhibited the migration of BT549R and 4T1‐LucR cells (Figure 7D). The proliferation of BT549R and 4T1‐LucR cells was higher than that of intolerant cells at a dose of 10 Gy, while aspirin decreased the proliferation of BT549R and 4T1‐LucR cells (Figure 7E). It can be concluded that a low dose of aspirin can promote the killing effect of radiotherapy on cancer cells and enhance the radiosensitivity of radioresistant BC cells.

### 3.8 Aspirin can enhance the sensitivity of BC to radiotherapy

**FIGURE 8:**
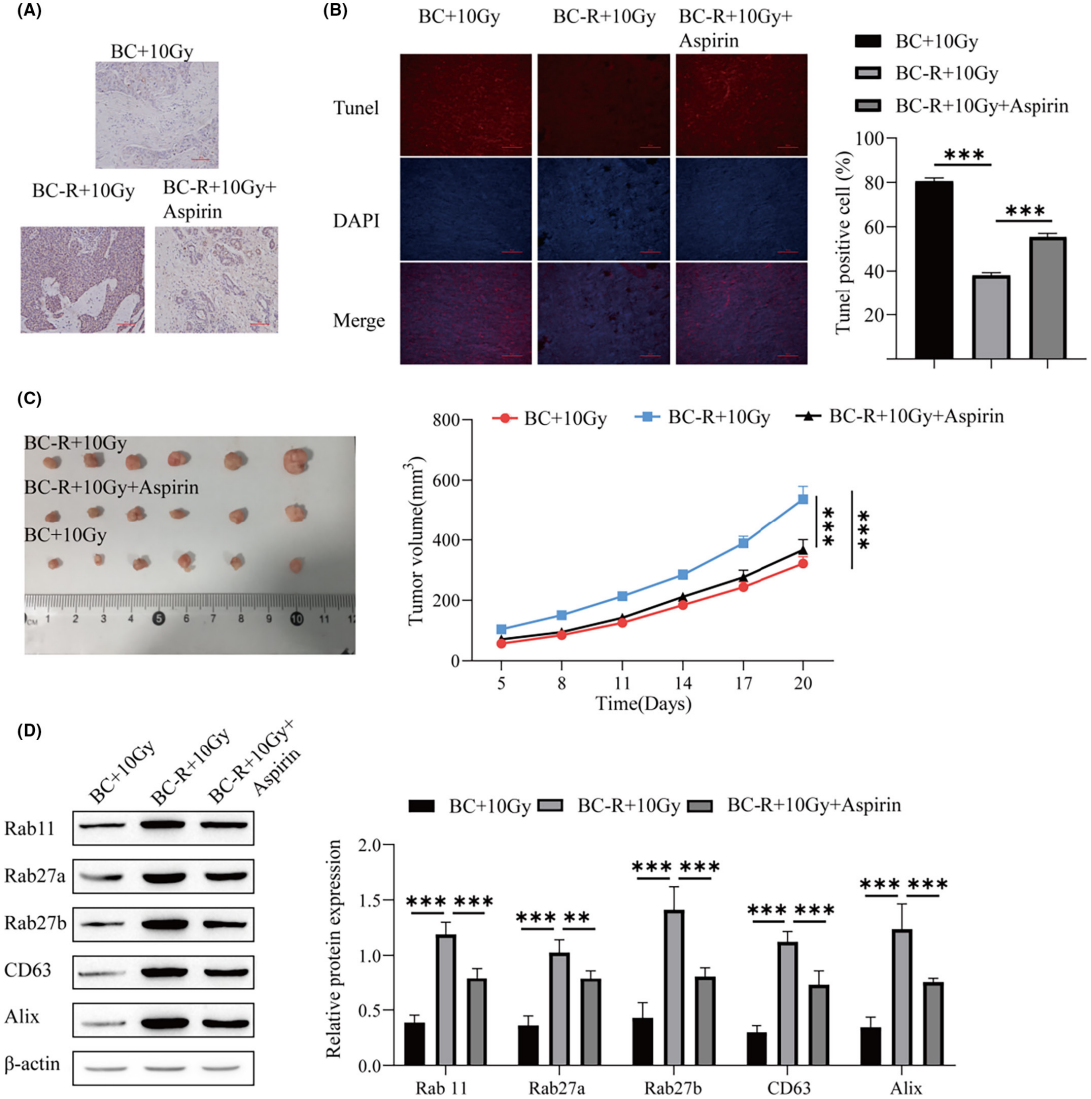
Aspirin can enhance the sensitivity of breast cancer (BC) to radiotherapy. (A) Immunohistochemistry was used to detect the expression of Ki‐67 in tissues. (B) TUNEL was used to detect apoptosis. (C) Tumor formation was detected in nude mice. (D) Western blotting was used to detect the protein levels of genes involved in exosomal biogenesis and secretion (Rab 11, Rab27a, Rab27b, CD63, and Alix transcripts). ***p < 0.001, **p < 0.01, *p < 0.05.

A low dose of aspirin can promote the killing effect of radiotherapy on cancer cells and enhance the radiosensitivity of radioresistant BC cells in vitro. Next, we will explore the effects of low doses of aspirin on tolerant BC cells in animals. BT‐549R cells were cultured first and then injected into nude mice according to the grouping. Nude mice were treated with 10 Gy radiotherapy and aspirin by gavage. Cell proliferation was observed by immunohistochemical detection of Ki‐67. The cell proliferation of the BC‐R + 10 Gy group was higher than that of the BC + 10 Gy group. Cell proliferation was lower in the BC‐R + 10 Gy + aspirin group than in the BC‐R + 10 Gy group (Figure 8A). TUNEL staining showed that apoptosis in the BC‐R + 10 Gy group was lower than that in the BC + 10 Gy group, and apoptosis in the BC‐R + 10 Gy + aspirin group was higher than that in the BC‐R + 10 Gy group (Figure 8B). The nude mouse tumorigenesis experiment showed that the tumor volume of the BC‐R + 10 Gy group was higher than that of the BC + 10 Gy group, and the tumor volume of the BC‐R + 10 Gy + aspirin group was lower than that of the BC‐R + 10 Gy group (Figure 8C). The influence of 10 Gy radiotherapy on tolerant BC cells was much less than that on intolerant BC cells, and the combination of radiotherapy with aspirin enhanced the sensitivity of tolerant BC cells. The protein levels of Rab11, Rab27a, Rab27b, CD63, and Alix in the BC‐R + 10 Gy group were significantly higher than those in the BC + 10 Gy group. Rab 11, Rab27a, Rab27b, CD63, and Alix protein expression was lower in the BC‐R + 10 Gy + aspirin group than in the BC‐R + 10 Gy group (Figure 8D). In conclusion, a low dose of aspirin can enhance the radiosensitivity of radioresistant BC cells and enhance the therapeutic effect of radiotherapy on BC.

## 4 DISCUSSION

Radiotherapy is the main treatment for BC, but some BC patients are not sensitive to radiation, which often leads to recurrence after treatment. Increasing radiosensitivity has become a difficult problem in the clinical treatment of BC.^38^ Some scholars have improved the efficacy of radiotherapy by improving the tumor microenvironment, scavenging free radicals and electrons, and inhibiting damage repair.^39^, ^40^ Our experiment investigated the radiosensitivity of aspirin by promoting radiation‐induced apoptosis of BC cells.

Aspirin is an antipyretic and analgesic drug with antipyretic, analgesic, and anti‐inflammatory effects. It is often used to treat colds, headaches, fever, and rheumatic diseases and to prevent cardiovascular diseases.^41^ However, current studies have found that aspirin not only has the above classical pharmacological effects but can also reduce the incidence and case fatality rate of rectal cancer, colon cancer, and other cancers, playing a key role in antitumor therapy, and its safety is higher than that of other antitumor drugs. Increasing attention has been given to the treatment of cancer patients with tumors.^42^ In CRC prevention studies, low doses of aspirin (75 mg/d) and high doses of aspirin (75–325 mg/d) were found to be equally effective in preventing CRC.^43^ Additional studies have shown that both low (81–160 mg/d) and high (300–325 mg/d) doses of aspirin reduced the risk of all adenomas and advanced adenomas.^44^ At present, there is no definite conclusion about the appropriate time for aspirin administration to prevent cancer risk, but the mainstream view is that aspirin needs to be used long‐term to reduce the risk of cancer and mortality rate.^45^ Considering the risk of gastrointestinal bleeding and other adverse reactions, it is more appropriate to use low‐dose aspirin to prevent cancer and reduce the risk of recurrence. The benefit of preventing the risk of cancer also increases significantly.^46^ To exclude the effect of aspirin itself on apoptosis and reduce the error of the experiment, we chose a concentration (2 mmol/L) that had no effect on cell proliferation to treat the cells. For aspirin, the results showed that there was increased radiation‐induced apoptosis of resistant cancer cells compared with the single irradiation group. In vivo, the tumor volume of nude mice in the BC‐R + 10 Gy group was larger than that of the BC + 10 Gy group, and the tumor volume of nude mice in the BC‐R + 10 Gy + aspirin group was lower than that of the BC‐R + 10 Gy group, which indicated that aspirin could increase the radiosensitivity of cancer cells.

Radiotherapy can directly or indirectly damage DNA and cause the death of tumor cells and can also attack immune cells to a certain extent.^46^ Some studies suggest that radiotherapy reduces the activity of immune cells through radiation damage to DNA, inhibits its killing effect on tumor cells, and inhibits the immune system of the body.^47^, ^48^ CD16 and CD56 are NK cell surface markers.^49^ First, we detected the expression of CD3, CD16, and CD56 by flow cytometry and found that CD3 was negative and CD16 and CD56 were positive, which indicated that NK cells were cocultured with exosomes. In the animal experiment to explore the inhibition of aspirin on the release of BC exosomes and the progression of BC, the expression of CD11b and CD27 protein was also detected by Western blot, and CD11b and CD27 are NK cell maturation markers.^50^ The expression of CD11b and CD27 protein was inhibited by 10 Gy radiotherapy, and aspirin attenuated the inhibitory influence of 10 Gy radiotherapy on NK cell proliferation. In addition, the radiation dose of 10 Gy not only caused the apoptosis of BT549 and 4T1‐Luc cells in vitro, but also reduced the tumor volume of nude mice in vivo, indicating that radiotherapy not only has a killing effect on BC cells but also has an inhibitory effect on the immune system.

Exosomes are pleomorphic vesicle‐like bodies secreted by various types of cells, most of which are between 30 and 100 nm in diameter. It contains many components, such as protein and RNA.^51^ Studies have shown that exosomes derived from senescent endothelial cells contain different proangiogenic miRNAs and proteins that can be used as biomarkers for age‐related vascular diseases.^52^ TEX, as miRNA information transmission carriers, promote the proliferation, metastasis, invasion and EMT of cocultured tumor cells, influencing the tumor microenvironment, enhancing tumor invasion and metastasis, mediating tumor immunosuppression and tumor resistance to radiotherapy and chemotherapy, ultimately leading to tumor metastasis.^53^, ^54^ After radiotherapy, chemotherapy, and other stimuli, exosomes carry information molecules to regulate communication and regulation between cells, and tumors produce more exosomes to accelerate the repair and generation of tumor blood vessels.^55^, ^56^ In this study, exosomes were extracted from BT549 and 4T1‐Luc cells treated with different radiotherapy conditions. Western blotting was used to detect the protein expression levels of genes associated with exosomal biogenesis and secretion (Rab 11, Rab27a, Rab27b, CD63, and Alix). CD63 is a positive marker for exosomes, and Rab11, Rab27a, and Rab27b are molecules associated with the exosome secretory pathway.^57^ The results showed that the protein expression of genes related to exosome formation and secretion in BT549 and 4T1‐Luc cells was significantly increased at a dose of 10 Gy compared with nonirradiated control cells. Exosomes secreted by BT549 and 4T1‐Luc cells after 10 Gy irradiation were detected by flow cytometry to stimulate NK cell apoptosis in vitro. Rab27a, a small GTPase that regulates multivesicular body–plasma membrane docking, can control exosomal secretion by inhibiting the proteasomal degradation of Rab27a.^58^ In this study, it was found that knockdown of Rab27a reduced the protein levels of genes involved in exosomal biogenesis and secretion in cancer cells and promoted the proliferative activity of aspirin on NK cells.

In summary, this study found that low doses of aspirin can inhibit the release of exosomes from BC cells induced by radiotherapy, reduce radiotherapy resistance in BC, weaken the viability of tumor cells, promote NK cell proliferation, strengthen immune supervision, and reduce the risk of cancer recurrence and metastasis. This study provides an important reference for the potential mechanism of BC development and the development of new therapeutic targets. As a treatment, aspirin can mediate the release of BC exosomes by inhibiting the expression of Rab27a as an effective drug to alleviate BC radiotherapy resistance. However, there are certain limitations in this study, which limit the ability to validate aspirin in clinical samples to improve the radiotherapy resistance of BC cells through the mediation of exosome release.

## AUTHOR CONTRIBUTIONS

**Li Wang:** Project administration (equal); writing – original draft (lead). **Zaoxiu Hu:** Project administration (equal); writing – original draft (lead). **Ceshi Chen:** Formal analysis (lead); methodology (lead); software (lead). **Ting Chen:** Data curation (lead); formal analysis (lead); software (lead). **Zhihong Yao:** Data curation (lead); investigation (lead); methodology (equal). **Wenhui Li:** Project administration (lead); writing – review and editing (lead). **Zuozhang Yang:** Funding acquisition (lead); project administration (lead); writing – review and editing (lead).

## FUNDING INFORMATION

This study was supported by the Basic Research Program of Science and Technology Department of Yunnan Province (202201AY070001‐142).

## CONFLICT OF INTEREST STATEMENT

The authors declare that they have no competing interests.

## ETHICS STATEMENT

All experimental mouse protocols were approved by the Ethics Review Committee of Animal Experiments of Kunming Medical University (kmmu20211083), and the animals were handled according to the management requirements of the Animal Management Association.

## Data Availability

The datasets used and/or analyzed during the current study are available from the corresponding author upon reasonable request.
